# Italian Tomato Cultivars under Drought Stress Show Different Content of Bioactives in Pulp and Peel of Fruits

**DOI:** 10.3390/foods11030270

**Published:** 2022-01-20

**Authors:** Veronica Conti, Marco Romi, Massimo Guarnieri, Claudio Cantini, Giampiero Cai

**Affiliations:** 1 Department of Life Sciences, University of Siena, 53100 Siena, Italy; marco.romi@unisi.it (M.R.); massimo.guarnieri@unisi.it (M.G.); giampiero.cai@unisi.it (G.C.); 2 National Research Council of Italy, Institute for Bioeconomy (CNR-IBE), 58022 Follonica, Italy; claudio.cantini@ibe.cnr.it

**Keywords:** abiotic stress, water stress, vitamin C, lycopene, rutin, caffeic acid, naringenin

## Abstract

Background: This study aims to evaluate the performance, in terms of accumulation of antioxidant compounds in fruits, of nine local and three commercial Italian tomato cultivars subjected to drought stress. The same local cultivars had been previously studied at morpho-physiological level. Methods: The present manuscript analyzes drought stress as a tool to increase the amount of secondary metabolites that can enhance fruit quality. Nutraceutical characterization of the fruits was performed by analyzing the content of antioxidants, phenols, flavonoids, lycopene, ascorbic acid (vitamin C), rutin, caffeic acid, and naringenin. At the same time, plant sensitivity to stress during the reproductive phase was monitored in terms of flower abscission, fruit drop, and seed germination. Results: Perina turns out to be the tomato cultivar with the best nutraceutical properties in the absence of stress while the Quarantino cultivar is so for flavonoid content (control plants) and lycopene and vitamin C content (stressed plants). Perina and Quarantino are the cultivars with the best response to drought and Perina has the highest concentrations of bioactives. Quarantino responds most effectively to stress in the reproductive phase. Conclusions: data confirm that drought stress increases bioactive production in some local cultivars of tomato, which produce higher quality fruits.

## 1. Introduction

Tomato fruits have good nutritional qualities as they contain active biomolecules and elements beneficial to human health, for example vitamin C, potassium, folic acid, carotenoids [1,2], polyphenols such as hydroxycinnamic acids (caffeic acid, chlorogenic acid) and flavonoids such as rutin, quercetin, and naringenin [3,4]. Indeed, many studies have linked the dietary consumption of tomatoes to the prevention and lower risk of cardiovascular and coronary heart disease, as well as cancer [5]. This protective action is attributed to secondary metabolites such as antioxidants, polyphenols, flavonoids, and anthocyanins [6]. Genetic factors, ripeness, and environmental conditions lead to differences in the biometabolic and nutraceutical characteristics of tomatoes [7]. Differences in biomolecule content have often been found between the exocarp, mesocarp, and endocarp of tomato fruits. Examples can be found in the cultivar Camone [4], where the peel contains the highest concentration of polyphenols while the mesocarp contains about four times less. The most abundant flavonoid in Camone is rutin, present in the peel. In another study, three commercial New Zealand tomatoes were shown to contain higher levels of polyphenols, flavonoids, lycopene, and ascorbic acid in the fruit peel than in the pulp and seeds [8].

Various stress conditions (including drought) can induce a significant increase in bioactive molecules. For plants, these molecules are of critical importance in the defense against abiotic stress [9]. In the case of drought, production of reactive oxygen species (ROS) or free radicals is a consequence of stress and leads to oxidative damage to proteins, DNA, and lipids [10]. Antioxidants have the function of scavenging free radicals and they include flavonoids, ascorbate, glutathione, carotenoids, and tocopherols [11].

However, the exposure to drought stress causes morphological, anatomical, physiological, and biochemical changes and, consequently, affects the growth and development of organs. Drought (as well as heat stress) damages the reproductive stage, leading to pollen sterility and reduced flower development with consequent decrease in seed and fruit production [12,13,14]. When drought stress occurs during seed formation, this leads to reduced seedling vigor and germination [15]. In crops, drought drastically reduces production and thus commercial performance [16]. Just to name a few examples, drought stress in sunflowers during germination compromises yield before the seeds even germinate [17,18]; in wheat, drought stress prior to flowering causes a decrease in grain number and size [19].

In tomato, drought stress significantly affects yield [20,21] as well as fruit volume, diameter, and composition in nutrients and biomolecules [22]. The tomato plant is sensitive to lack of water during reproductive development, especially during flowering and fruit growth [23]. Under drought stress conditions, tomato plants exhibit reduced leaf area and growth, flower drop, mineral deficiency, reduced fruit size, fruit breakage, and calcium deficiency-related physiological disorders such as flower rot and poor seed viability [24].

Today, a more sustainable agriculture, which requires fewer water resources, must take into account genetic biodiversity as a fundamental factor for improving yield and quality of crops, as well as resistance to biotic and abiotic stress. In the long term, this would allow farmers to sustain productivity even in drastic environmental conditions. This requires the identification and use of species/cultivars best adapted to their growing area. Indeed, local cultivars are a source of unique genetic traits derived from adaptation to their area of origin, are often more resistant to biotic and abiotic stresses, and have high content of phytochemicals beneficial to human health [25,26,27]. In Italy, several tomato cultivars are present, adapted to growing environments and selected for agronomic traits of interest, such as productivity, transportation durability, and marketability [28]. Italy, and specifically the Tuscany region, is also characterized by locally adapted cultivars that show marked genetic variability (compared to commercial cultivars) [29].

In previous works [29,30], we have analyzed locally adapted Tuscan tomato cultivars to prove their tolerance to water deficiency while identifying the most tolerant and susceptible. The previous data at morphological and physiological levels have allowed us to catalog the Tuscan cultivars based on their resistance to drought. However, analyses stopped at the vegetative and reproductive phase without considering the phytochemical content of fruits. The content of bioactive compounds in fruit pulp and peel was correlated with seed set and the development of flowers and fruits, to get an indication of the susceptibility to drought stress and to highlight the most promising cultivars (both under stress and non-stress conditions) in terms of nutraceutical compounds.

The starting hypothesis is that tomato peel extracts represent a reliable source of bioactive molecules that can protect human health from oxidative stress [31]. In this work, we evaluated whether locally adapted (and drought stressed) Tuscan tomato cultivars can biosynthesize more antioxidant compounds in fruits. Thus, the tomato defense mechanism could be exploited to increase the production of secondary antioxidant metabolites useful for human health.

## 2. Materials and Methods

### 2.1. Plant Growth and Drought Conditions

Cultivars of *Solanum lycopersicum* L. were provided by the Tuscan regional germplasm bank. The nine cultivars are “Canestrino di Lucca”, “Costoluto Fiorentino”, “Fragola”, “Giallo di Pitigliano”, “Perina a Punta della Valtiberina”, “Pisanello”, “Quarantino ecotipo Valdarno”, “Rosso di Pitigliano”, “Tondino Liscio da Serbo Toscano”. To these we added, “Datterino”, “Pantano” and “Pearson”, three commercial cultivars found in the large-scale distribution. The commercial cultivars were chosen because of their morphological similarity with some local Tuscan cultivars and because they are marketed in Tuscany as well as in Italy.

Seeds of each cultivar were germinated in Petri dishes on filter paper soaked with distilled water at a constant temperature of 25 °C. Afterwards, seedlings were transferred to a greenhouse (Botanical Garden, University of Siena) and planted in a tray with wells (4 × 5 × 6 cm) at 25 °C. For each cultivar, eight plants were studied during the reproductive growth phase; plants were transferred into square PE pots (upper diameter of 28 cm, base diameter of 22 cm, 24 cm high). Plants were divided into two groups: four plants were subjected to drought stress (DS) while four were the controls (CTRL) [32]. All plants were positioned in the greenhouse according to a randomization plan, with temperature and humidity monitored by a datalogger EBI 20-TH1 (ebro^®^), the daily mean and standard deviation were computed separately for day and night hours. The mean temperature and humidity in daytime hours were 32.7 ± 3.8 °C and 50.7 ± 8.4%, respectively; during nighttime hours, the mean temperature was 23.9 ± 2.1 °C while the mean humidity was 64.7 ± 3.2%. The CTRL group was irrigated regularly, while the DS group was subjected to a total lack of water for 20 days. The DS treatment was based on existing literature [33,34]. Fruits, when fully ripe (total red fruit), were sampled and stored at −80 °C.

### 2.2. Development of Flowers and Fruits

**Figure 1:**
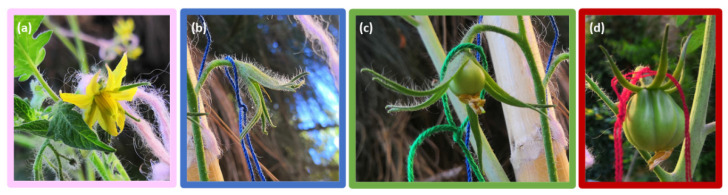
An example of marking made at various developmental stages on the same plant: (**a**) pink for opening flowers, (**b**) blue for fertilized flowers, (**c**) green for small green tomatoes, and (**d**) red for large unripe green tomatoes.

To study flower development during drought stress, at the beginning of stress (t_0_) plants of each cultivar were marked with differently colored strings (pink for open flowers, blue for fertilized flowers, green for small green tomatoes (period of cell division), red for large green unripe tomatoes (period of cell expansion close to the ripening period), which are shown in Figure 1), these phases-marking steps were taken from Azzi [35] and Mazzucato [36]. The temporal development of each flower and fruit was monitored by counting them at the middle (10 days, t_1_) and end (20 days, t_2_) of drought stress. For each marker, counts made at t_1_ and t_2_ were reported as a percentage of those at t_0_.

### 2.3. Germination of Seeds

Seeds were removed from three fruits of each cultivar, washed with water, dried on tissue paper, and then stored in polyethylene bags at room temperature. The germination test was performed by placing 100 seeds on two layers of moist filter paper in Petri dishes. Seed germination was calculated daily for eight days. A seed was considered germinated when a 3–4 cm long rootlet was visible outside the seed coating [15]. Percentage of germination and shoot length were recorded.

### 2.4. Preparation of Samples for Colorimetric Analysis

For each tomato cultivar, five fruits were selected randomly and chopped. Then, 1 g of peel and 1 g of pulp were weighed and 6 mL (for peel) and 3 mL (for pulp) of 70% acetone were immediately added. Samples were homogenized by Turrax (Ultra-Turrax^®^ T25 based IKA, Saint Louis, MO, USA) for 5 min, then placed in a sonicator (Elma Transsonic T 460/H, Wezikon, Switzerland) for 15 min and then homogenized again by Turrax. Samples were then centrifuged at 4000× *g* r.p.m. for 5 min (Eppendorf^®^ 5415D centrifuge, Hamburg, Germany). Finally, supernatants (i.e., the extract) were transferred to 2 mL Eppendorf tubes.

### 2.5. Determination of the Antioxidant Power

The total antioxidant potential of tomato peel and pulp extracts was determined using the FRAP (ferric reducing antioxidant power) assay reported by Benzie and Strain [37]. The test is based on reduction of Fe3+-2,4,6-Tri (2-pyridyl)-s-triazine (TPTZ) to a blue Fe2+-TPTZ. The absorbance was read at 593 nm (Perkin Elmer spectrophotometer, Lamba 25, Waltham, MA, USA). The FRAP value of extracts, expressed as µmol Fe2+/g of fresh weight (FW), was determined using a standard curve of ferrous sulphate. The experiment was conducted in three technical replicates for each sample. Finally, the mean and standard deviation were calculated. To verify the significance of the data obtained, the *t*-test (* *p* ≤ 0.05, ** *p* ≤ 0.01) were carried out.

### 2.6. Determination of Phenolic Content

The total polyphenol content (TPC) of tomato peel and pulp extracts was determined in fruits by the spectrophotometric method of Folin–Ciocâlteu [38]. This assay is based on electron transfer in alkaline medium from phenolic compounds to phosphomolybdic/phosphotungstic acid complexes, which are read at 765 nm. Results were expressed in gallic acid equivalent (GAE), a universally accepted standard for polyphenols, to determine the value of TPC in mg/100 g of fresh weight (FW). Actually, the reagent used in the Folin–Ciocâlteu method is not strictly specific to phenolics and can react with other substances. Therefore, the results of the assay should more generally be interpreted as an estimate of the reducing capacity. The experiment was conducted in three technical replicates for each sample. Finally, the mean and standard deviation were calculated. To verify the significance of the data obtained, the *t*-test (* *p* ≤ 0.05, ** *p* ≤ 0.01) were carried out.

### 2.7. Determination of Flavonoid Content

Flavonoids are determined by the aluminum chloride assay. Complexes of aluminum chloride with flavonoids cause the solution to turn yellow, which is read by a spectrophotometer at 415 nm [39]. The data obtained were compared to a calibration curve obtained with the quercetin standard. Values were expressed as mg of total flavonoids in 100 g of fresh weight (FW). The experiment was conducted in three technical replicates for each sample. Finally, the mean and standard deviation were calculated. To verify the significance of the data obtained, *t*-tests (* *p* ≤ 0.05, ** *p* ≤ 0.01) were carried out.

### 2.8. Determination of Lycopene

Extraction of lycopene was made according to Barba [40]; 0.3 g of tomato peel and pulp (taken from the pull described previously in Section 2.4) were added to 10 mL of a solvent solution made by hexane/acetone/ethanol (50:25:25 *v*/*v*/*v*) and homogenized with Ultra-Turrax (IKA^®^). Subsequently, 1.5 mL of distilled water was added, and the samples were vortexed. The upper layer (1 mL) was dried under vacuum and the dry extract was resuspended in 0.4 mL of tetrahydrofuran (THF)/acetonitrile (ACN)/methanol (15:30:55 *v*/*v*/*v*). The mobile phase for HPLC (Perkin Elmer Nelson 3200 Series) analysis consisted of methanol/ACN (90:10 *v*/*v*) and 9 mM triethanolamine (TEA) at a flow rate of 0.9 mL/min, using a RP-C18 column (SUPELCO Kromasil 100A-5u-C18 4.6 mm × 250 mm); the absorbance was set at 475 nm and the run time was 20 min. Quantification was carried out using a standard calibration curve consisting of five points at increasing concentrations (6.25, 12.5, 25, 50, and 100 µg/mL) of lycopene standard (Sigma Chemical, St. Louis, MO, USA). The experiment was conducted in three technical replicates for each sample. Finally, the mean and standard deviation were calculated. To verify the significance of the data obtained, *t*-tests (* *p* ≤ 0.05, ** *p* ≤ 0.01) were carried out.

### 2.9. Determination of Vitamin C

Extraction of ascorbic acid was carried out using 1 g of both tomato peel and pulp (taken from the pull described previously in Section 2.4) in 2 mL of distilled water; samples were homogenized with Ultra-Turrax (IKA^®^), then filtered through a 0.45-µm membrane filter [41]. For HPLC analysis, an RP-C18 column (SUPELCO Kromasil 100A-5u-C18 4.6 mm × 250 mm) was used. The mobile phase consisted of 0.01 mol/l KH2PO4 buffer solution (pH = 2.6 with o-phosphoric acid), with a flow rate of 0.5 mL/min and an absorbance set at 250 nm. The quantification was carried out using a standard calibration curve consisting of five points at increasing concentrations (6.25, 12.5, 25, 50, and 100 µg/mL) of ascorbic acid standard (Sigma Chemical, St. Louis, MO, USA). The experiment was conducted in three technical replicates for each sample. Finally, the mean and standard deviation were calculated. To verify the significance of the data obtained, *t*-tests (* *p* ≤ 0.05, ** *p* ≤ 0.01) were carried out.

### 2.10. Determination of Rutin, Quercetin, Naringenin, and Caffeic Acid

Determination of rutin, quercetin, naringenin, and caffeic acid was performed with an RP-C18 column (SUPELCO Kromasil 100A-5u-C18 4.6 mm × 250 mm). Sample extraction was performed according to Tokusoglu [42], with some modifications. Samples of peel and pulp fruit (1 g, taken from the pull described previously in Section 2.4) were added to 1 mL of 70% acetone containing 1% (*v*/*v*) HCl and 0.02 mg/mL TBHQ (tert-Butylhydroquinone). The mixture was then homogenized by Ultra-Turrax (IKA^®^) and 0.2 mL of 1.2 M HCl was added. The mixture was incubated at 90 °C for 2 h under continuous stirring. Samples were then cooled at room temperature and sonicated for 3 min. Finally, extracts were centrifuged for 5 min at 3000× *g* and filtered through a 0.45-µm membrane filter. The HPLC method was performed according to Kumar [43], with slight modifications. The mobile phase was water (phase A) and acetonitrile with 0.02% trifluoracetic acid (TFA) (phase B); elution was performed with a linear gradient of 80% A and 20% B (0–5 min), 60% A and 40% B (5–8 min), 50% A and 50% B (8–12 min), 60% A and 40% B (12–17 min), 80% A and 20% B (17–21 min). The flow rate was 1 mL/min, and the absorbance was set at 365 nm for rutin and quercetin, 325 nm for caffeic acid, and 280 nm for naringenin; the run time was 21 min. Quantification was carried out using standard calibration curves consisting of five points from 5 to 80 µg/mL using standards of rutin, quercetin, naringenin, and caffeic acid (Sigma Chemical, St. Louis, MO, USA). The experiment was conducted in three technical replicates for each sample. Finally, the mean and standard deviation were calculated. To verify the significance of the data obtained, *t*-tests (* *p* ≤ 0.05, ** *p* ≤ 0.01) were carried out.

## 3. Results

### 3.1. Development of Flowers and Fruits

Analysis of flower and fruit development throughout the drought stress period provided an indication of the reproductive (and thus productive) performance of plants [44]. In general, drought stress induces early flowering, which could be due to rapid phenological development aimed at completing the life cycle under unfavorable environmental conditions. The results obtained on the development of open flowers (pink thread) reveal that in most cultivars the loss of open flowers is less than 50% both for the CTRL and DS groups (Figure S1). Major differences are found between CTRL and DS in cultivars such as Giallo, Perina, and Pisanello, where loss is higher in DS samples with 57% total loss compared to 10% in the CTRL group. By contrast, in cultivars such as Tondino and Rosso the loss in the DS group is less than the CTRL group or even no loss at all. Lower ratio of abscised flowers in tolerant genotypes could also be due to maintenance of efficient photosynthesis [22]. Indeed, reduced photosynthesis decreases the availability of sugars and their contribution to floral organ development leading to their abscission [45,46]. Development of fertilized flowers (blue thread) shows a general delay in the DS group compared to the CTRL, and loss is always higher in the DS. An exception is the cultivar Perina for which both loss and development time are comparable to the CTRL group. In contrast, in the cultivars Rosso, Datterino, and Pantano flowers in the DS group develop earlier, whereas for Datterino there is substantial loss in the DS group compared to CTRL. In contrast, there is no fruit drop in the stages marked with green wires, i.e., those that monitored small green fruits. However, for the cultivars Rosso, Tondino, and Fragola, development is delayed in the DS groups compared to the CTRL; the cultivar Costoluto shows early development in the DS group while the cultivar Perina exhibits development similar to the CTRL group. Red thread (marking the growth and ripening of large green fruits) does not reveal major losses in plants subjected to drought stress. In most cultivars there is early ripening in the DS group, but for the cultivars Costoluto, Giallo, Perina, Datterino, and Pearson, development times in the DS group are remarkably similar to the CTRL group.

### 3.2. Seed Germination

**Figure 2:**
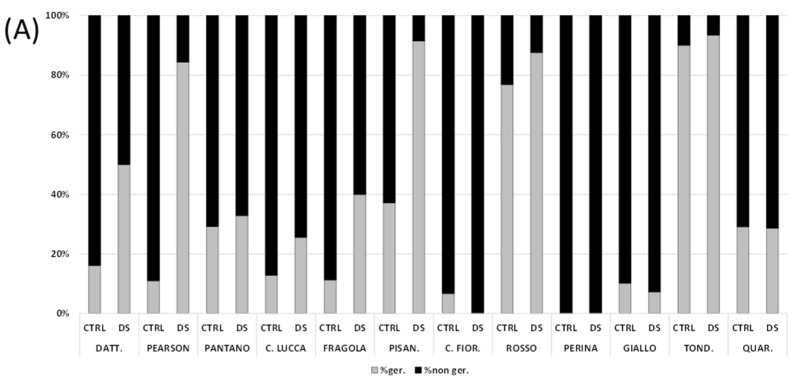

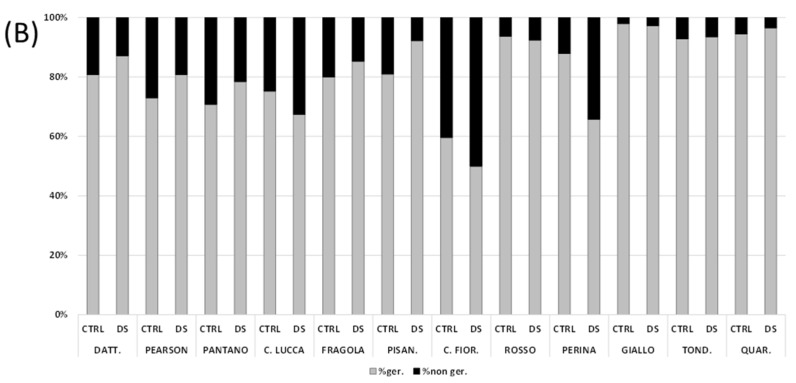
(**A**) Seed germination after 4 days expressed in percentage. (**B**) Seed germination after 8 days expressed in percentage. In black the percentage of non-germinated seeds and in gray the percentage of germinated seeds. CTRL indicates the control group of tomato, and DS the drought-stressed group.

Seed germinability is an index of the productivity and reproductive efficiency of plants. This aspect was tested to monitor the effect of drought stress on the production capacity of the cultivars under examination. Differences between the cultivars were already visible after 4 days (Figure 2A). The Datterino, Pearson, Fragola, and Pisanello cultivars show a clear progress in the germination of seeds from tomatoes that suffered stress. This does not occur in the case of seeds of control tomatoes. The Pantano, Canestrino di Lucca, Rosso, and Tondino cultivars show a progress of germination in seeds from stressed plants, but the performance remains similar to that of controls. In the Quarantino cultivar, after 4 days no differences in the germination rate between control seeds and seeds of stressed plants are observed. On the contrary, the germination rate of the Costoluto Fiorentino and Giallo cultivars is low for both CTRL and DS. The Perina cultivar shows no differences because after 4 days neither the CTRL seeds nor the DS seeds are germinated.

After 8 days, the germination rate is adequate for all cultivars but with some differences (Figure 2B). Datterino, Pearson, Pantano, Fragola, and Pisanello cultivars exhibit a fair percentage of germination in both the CTRL and DS; for the latter the percentage is slightly higher, probably because of early germination of seeds. The opposite occurs for Canestrino di Lucca, Costoluto Fiorentino, and Perina in which the percentage of germinated seeds of the DS group is lower than the CTRL. The highest germination rate in CTRL is found in Perina. Rosso, Giallo, Tondino, and Quarantino cultivars show a nearly equal germination rate between CTRL and DS.

### 3.3. Antioxidant Power in Peel and Pulp

**Figure 3:**
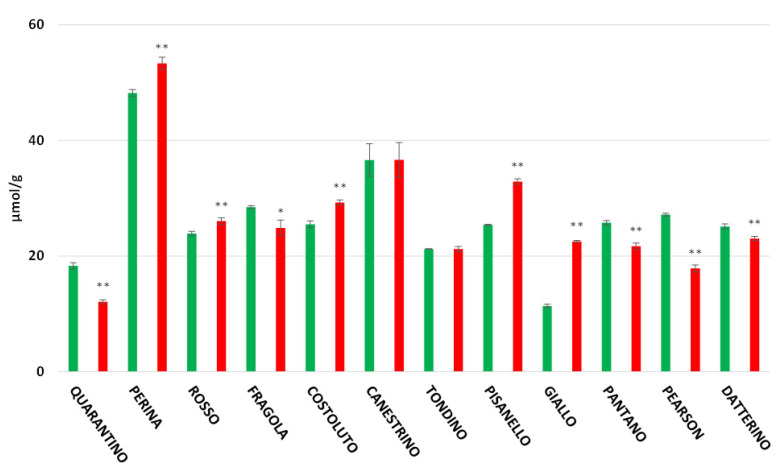
Total antioxidant (expressed as µmol/g of fresh weight (FW)) in tomato peel of 9 Italian cultivars and three commercial cultivars. Controls (CTRL) in green and drought-stressed group (DS) in red. The bars indicate the standard deviation. A significantly difference is shown between CTRL and DS of each cultivar by * for a *p* ≤ 0.05 and ** for *p* ≤ 0.01.

Analysis of the peel (Figure 3) showed that stressed plants of the three commercial cultivars exhibit a decrease in antioxidant power compared to controls. The three commercial cultivars have similar values: in stressed plants the antioxidant power is around 20 μmol/g, while in the control group it is around 25 μmol/g. More precisely, the stressed Pearson cultivar has the lowest value (17.86 μmol/g) and undergoes a drastic decrease compared to the control (27.17 μmol/g). Among local cultivars, Perina has the highest antioxidant power for both the CTRL group (48.19 μmol/g) and the DS group (53.30 μmol/g). In this case, the antioxidant capacity in the peel of stressed plants is higher than in control plants. The cultivar Quarantino, on the other hand, has the lowest value under drought stress (12.10 μmol/g), a value below that of the CTRL group (18.32 μmol/g). Finally, the cultivar that most clearly increases the antioxidant content in the peel under drought stress is Giallo di Pitigliano, which shows an extremely low content in the control group (11.37 μmol/g) but it doubles in the stressed group (22.54 μmol/g).

**Figure 4:**
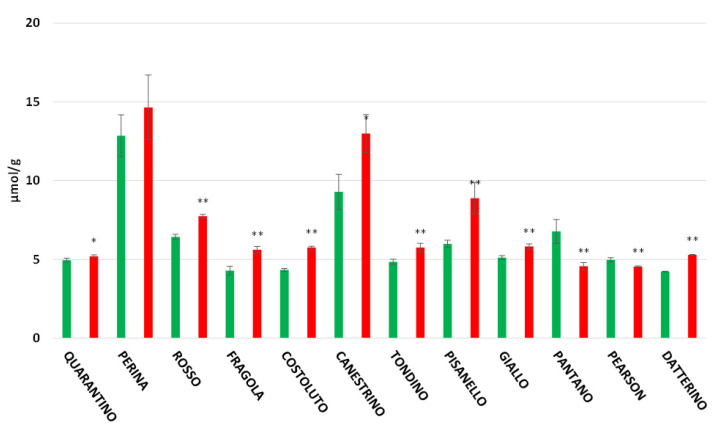
Total antioxidant (expressed as µmol/g of FW) in tomato pulp of 9 Italian cultivars and three commercial cultivars. Controls (CTRL) in green and drought-stressed group (DS) in red. The bars indicate the standard deviation. A significantly difference is shown between CTRL and DS of each cultivar by * for a *p* ≤ 0.05 and ** for *p* ≤ 0.01.

The overall picture in the pulp (Figure 4) remains the same as in the peel, with antioxidant contents being significantly lower in the pulp. The cultivar Perina has the highest antioxidant power in both the control (12.85 μmol/g) and the stressed group (14.65 μmol/g). A difference from the peel is observed for the cultivars Quarantino and Datterino, which have higher antioxidant content in the pulp in the CTRL group than in the DS.

### 3.4. Polyphenol Content

**Figure 5:**
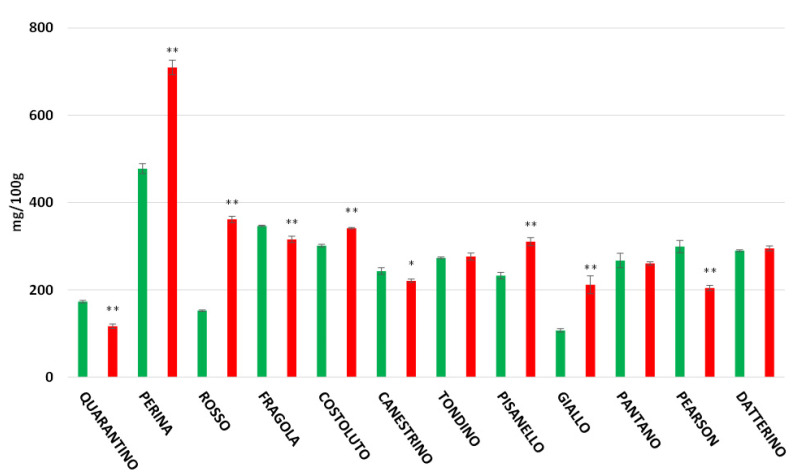
Total polyphenol content (expressed as mg/100 g of FW) in tomato peel of 9 Italian cultivars and three commercial cultivars. Controls (CTRL) in green and drought-stressed group (DS) in red. The bars indicate the standard deviation. A significant difference is shown between CTRL and DS of each cultivar by * for a *p* ≤ 0.05 and ** for *p* ≤ 0.01.

**Figure 6:**
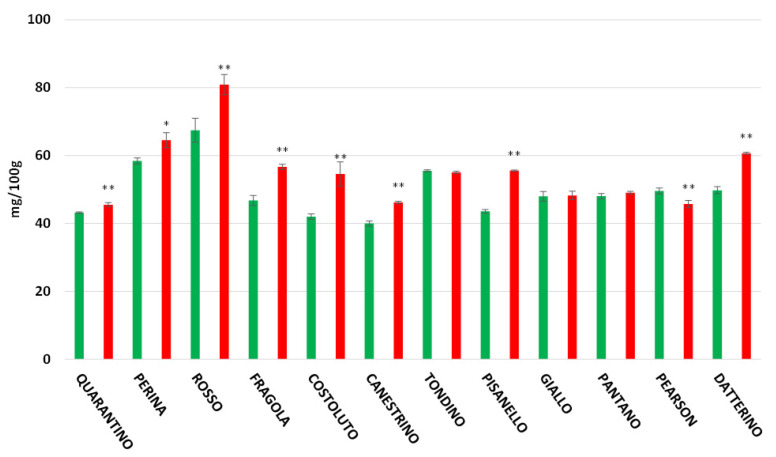
Total polyphenol content (expressed as mg/100 g of FW) in tomato pulp of 9 Italian cultivars and three commercial cultivars. Controls (CTRL) in green and drought-stressed group (DS) in red. The bars indicate the standard deviation. A significantly difference is shown between CTRL and DS of each cultivar by * for a *p* ≤ 0.05 and ** for *p* ≤ 0.01.

In the peel (Figure 5), the highest polyphenol content is found in the cultivar Perina (709.44 mg/100 g in the DS group and 477.77 mg/100 g for the CTRL group). The lowest content among stressed plants is found in the cultivar Quarantino (116.56 mg/100 g), where the amount of polyphenols is reduced if compared to when plants are hydrated (172.85 mg/100 g). The opposite situation occurs for Rosso di Pitigliano, which significantly increases polyphenol content under drought stress (361.33 mg/100 g) compared to the control (152.47 mg/100 g). In the pulp (Figure 6), the cultivar with the highest polyphenol content is Rosso di Pitigliano (80.88 mg/100 g for DS group plants and 67.46 mg/100 g for CTRL). The cultivar Perina maintains high values in both DS and CTRL groups.

### 3.5. Flavonoids Content

**Figure 7:**
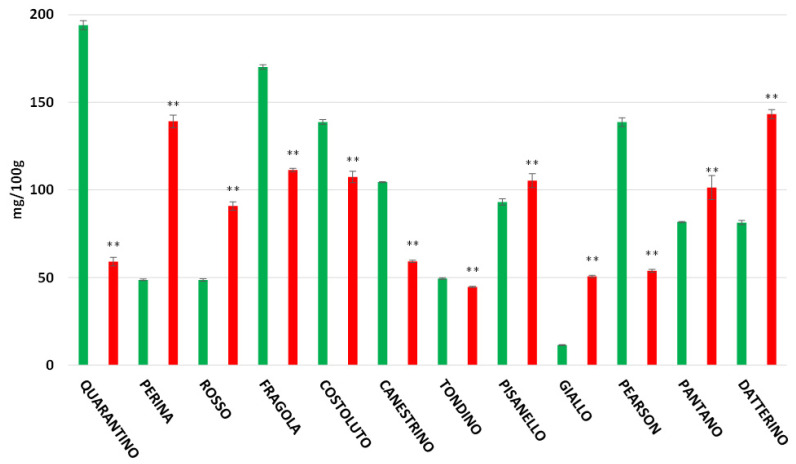
Total flavonoids content (expressed as mg/100 g of FW) in tomato peel of 9 Italian cultivars and three commercial cultivars. Controls (CTRL) in green and drought-stressed group (DS) in red. The bars indicate the standard deviation. A significantly difference is shown between CTRL and DS of each cultivar by ** for *p* ≤ 0.01.

**Figure 8:**
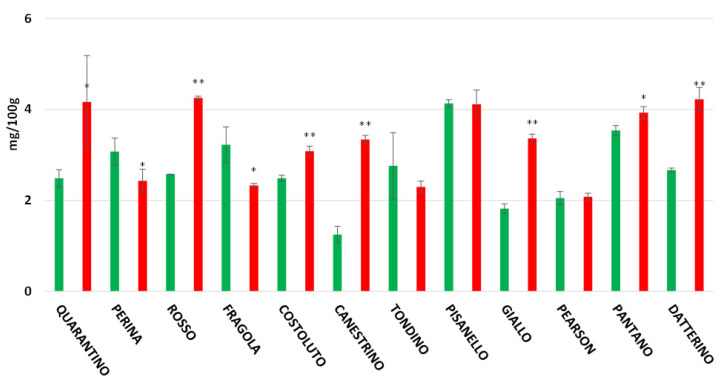
Total flavonoids content (expressed as mg/100 g of FW) in tomato pulp of 9 Italian cultivars and three commercial cultivars. Controls (CTRL) in green and drought-stressed group (DS) in red. The bars indicate the standard deviation. A significantly difference is shown between CTRL and DS of each cultivar by * for a *p* ≤ 0.05 and ** for *p* ≤ 0.01.

The highest flavonoid content recorded in the peel is found in the CTRL group of the cultivar Quarantino with 193.98 mg/100 g (Figure 7), a value that far exceeds the content of the corresponding stressed group (59.09 mg/100 g). The cultivars Costoluto, Canestrino, Fragola, and the commercial Pantano follow the same trend. In contrast, the cultivars Giallo, Perina, Pisanello, Rosso, Datterino, and Pearson showed an increase in drought-stressed plants. The highest flavonoid content for stressed plants was found in the cultivars Perina and Datterino, with about 140 mg/100 g. The stressed Perina cultivar had a particularly high flavonoid content compared to the control (48.67 mg/100 g). For the pulp, results are different (Figure 8). Stressed cultivars such as Costoluto, Canestrino, Giallo, Rosso, Datterino, and Pearson show increased flavonoid content compared to the control. Pisanello, Tondino, and Pantano cultivars show no clear differences between plants in the CTRL and DS groups. Fragola and Perina are the only two cultivars showing a decrease in stressed plants compared to controls.

### 3.6. Vitamin C

**Figure 9:**
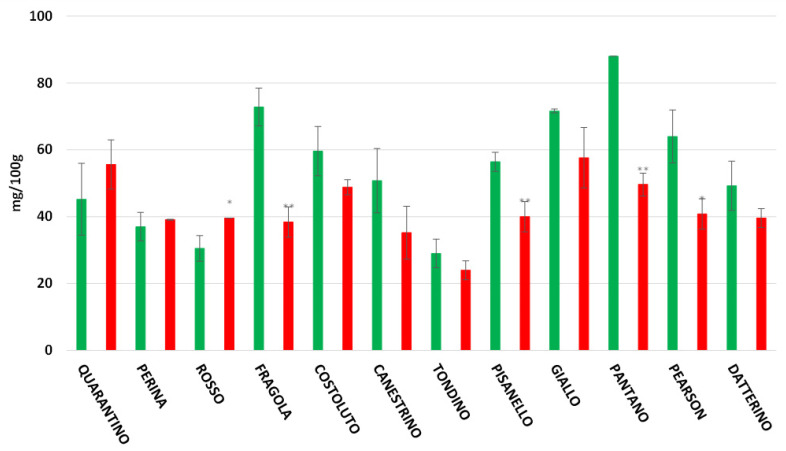
Vitamin C (ascorbic acid) content in tomato peel of 9 Italian cultivars and three commercial cultivars. Controls (CTRL) in green and drought-stressed group (DS) in red. The bars indicate the standard deviation. A significantly difference is shown between CTRL and DS of each cultivar by * for a *p* ≤ 0.05 and ** for *p* ≤ 0.01.

**Figure 10:**
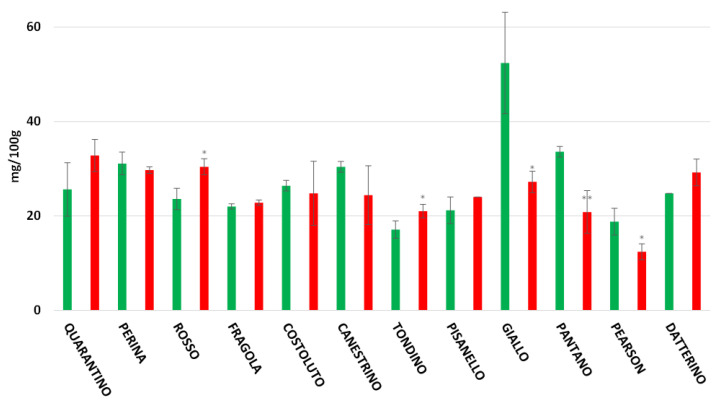
Vitamin C (ascorbic acid) content in tomato pulp of 9 Italian cultivars and three commercial cultivars. Controls (CTRL) in green and drought-stressed group (DS) in red. The bars indicate the standard deviation. A significantly difference is shown between CTRL and DS of each cultivar by * for a *p* ≤ 0.05 and ** for *p* ≤ 0.01.

The content of ascorbic acid in the skin of different cultivars is shown in Figure 9. For most genotypes, stressed fruits have lower vitamin C content than controls. This is particularly evident for Fragola, Pisanello, Giallo, Pantano, and Pearson. For other cultivars, such as Quarantino, Perina, Tondino, and Datterino, the ascorbic acid content of stressed fruits is similar to that of controls. On the other hand, the cultivar Rosso has a slightly higher content in stressed fruits than the control. In contrast, a few differences are found in the pulp (Figure 10). The stressed Rosso cultivar increases the content of vitamin C, as in the peel, and the Datterino cultivar behaves similarly. Giallo, Pantano, and Pearson decrease the ascorbic acid content in the pulp of stressed fruits just as in the peel. The concentration of vitamin C in the control of Giallo cultivar differs because the value in the pulp is also comparable to those in the peel.

### 3.7. Lycopene

**Figure 11:**
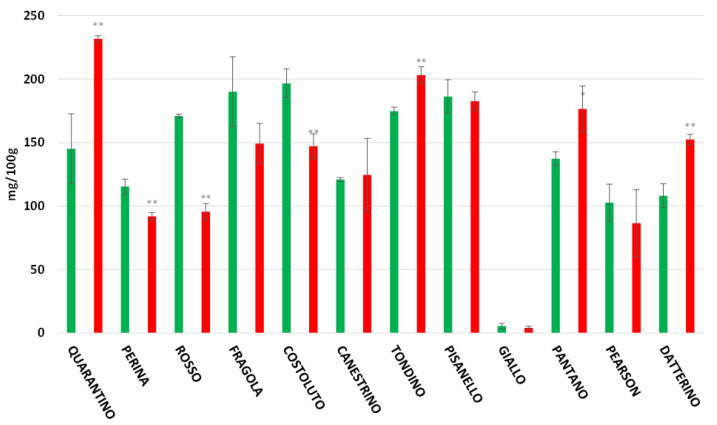
Lycopene content in tomato peel of 9 Italian cultivars and three commercial cultivars. Controls (CTRL) in green and drought-stressed group (DS) in red. The bars indicate the standard deviation. A significantly difference is shown between CTRL and DS of each cultivar by * for a *p* ≤ 0.05 and ** for *p* ≤ 0.01.

**Figure 12:**
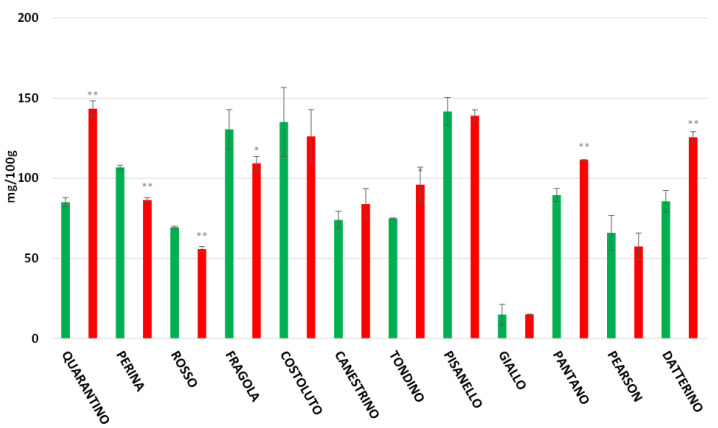
Lycopene Content in tomato pulp of 9 Italian cultivars and three commercial cultivars. Controls (CTRL) in green and drought-stressed group (DS) in red. The bars indicate the standard deviation. A significantly difference is shown between CTRL and DS of each cultivar by * for a *p* ≤ 0.05 and ** for *p* ≤ 0.01.

Lycopene is the most common carotenoid present in tomatoes. In the peel (Figure 11) the concentration is extremely high for all genotypes except for the Giallo cultivar. This was already inferred from the yellow color of its fruits, since higher amounts of lycopene provide a reddish color. The cultivars Quarantino, Tondino, Pantano, and Datterino show an increase in lycopene concentration in stressed fruits compared to controls. The opposite occurs for Perina, Rosso, and Costoluto. In the other cultivars, there are no significant differences between CTRL and DS. In the pulp, lycopene concentration is generally lower than in the peel, except for the cultivar Giallo, which conversely shows a higher content for both CTRL and DS (Figure 12). The cultivars Quarantino, Tondino, Pantano, and Datterino show an increase in lycopene in the pulp of stressed fruits as well as in the peel. The opposite occurs for Perina, Rosso, and Fragola. In all other cultivars there are no differences in lycopene concentration between CTRL and DS.

### 3.8. Rutin, Caffeic Acid and Naringenin

**Figure 13:**
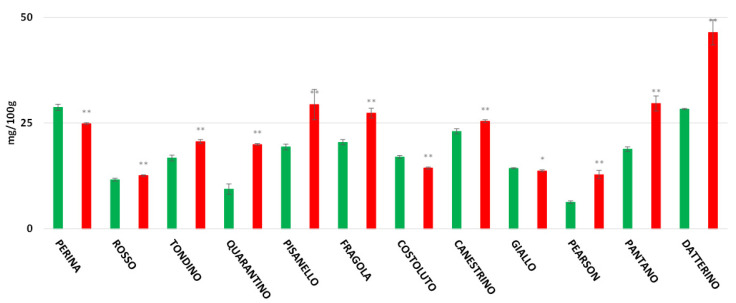
Rutin content in tomato peel of 9 Italian cultivars and three commercial cultivars. Controls (CTRL) in green and drought-stressed group (DS) in red. The bars indicate the standard deviation. A significant difference is shown between CTRL and DS of each cultivar by * for a *p* ≤ 0.05 and ** for *p* ≤ 0.01.

Rutin is the flavonoid most found in tomatoes [3,4]. Chemically, it is a glycoside composed of the flavonol quercetin aglycone and the disaccharide rutinose. In this study, rutin was not identified in the pulp, while it was found in high amounts in the peel of all cultivars (Figure 13). These showed an increase in the concentration of rutin in the peel of stressed tomatoes, except for the cultivars Perina and Costoluto in which the stressed peel underwent a decrease in concentration. In the cultivars Rosso and Giallo there are no clear differences between CTRL and DS. Quercetin was not found in any of the considered samples.

**Figure 14:**
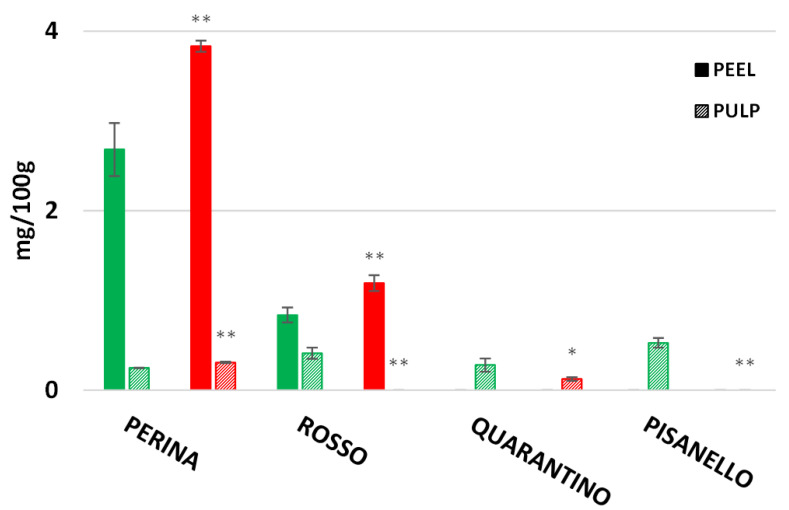
Caffeic acid content in 4 Italian tomato cultivars. The concentration of peel in full color and pulp in stripes color. CTRL indicates the control group of tomato and DS the drought-stressed group. The bars indicate the standard deviation. A significant difference is shown between CTRL and DS of each cultivar by * for a *p* ≤ 0.05 and ** for *p* ≤ 0.01.

Caffeic acid is part of the hydroxycinnamic acids and belongs to the family of polyphenols. In the present work caffeic acid was found only in four cultivars: Perina, Rosso, Quarantino, and Pisanello (Figure 14). High concentrations were found in the peel of Perina and Rosso cultivars, with higher values in drought-stressed plants. Small concentrations were instead found in the pulp of the cultivar Perina, both CTRL and DS, while in the cultivar Rosso caffeic acid was found only in the pulp of the CTRL group. In the cultivar Quarantino caffeic acid was found only in the pulp with lower contents in the DS group than in the CTRL. The cultivar Pisanello contains caffeic acid only in the pulp of control fruits while it is absent in stressed fruits.

**Figure 15:**
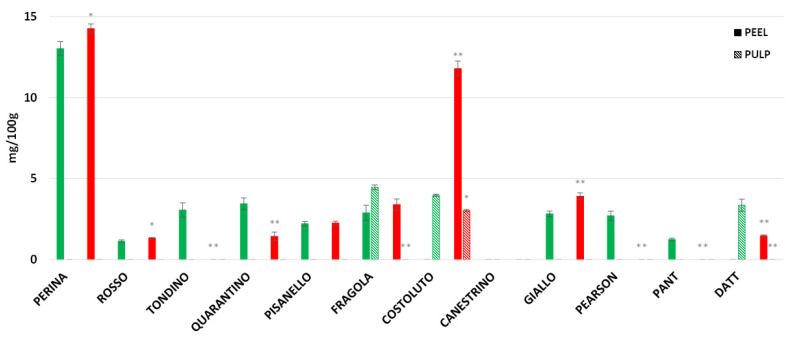
Naringenin content in tomato of 9 Italian cultivars and three commercial cultivars. The concentration of peel in full color and pulp in stripes color. CTRL indicates the control group of tomato and DS the drought-stressed group. The bars indicate the standard deviation. A significant difference is shown between CTRL and DS of each cultivar by * for a *p* ≤ 0.05 and ** for *p* ≤ 0.01.

Naringenin is a flavanone belonging to the flavonoid family. Figure 15 shows that in most cultivars, naringenin is present only in the fruit peel. The cultivars Fragola, Costoluto, and the commercial Datterino are the only cultivars that also show naringenin in the pulp. While the Fragola and Datterino cultivars show the presence of this flavonoid only in the pulp of control fruits, Costoluto contains naringenin also in the pulp of stressed fruits. The highest content of naringenin is present in the peel of cultivar Perina, both CTRL and DS, with an increase in the peel of stressed fruits, as well as for cultivar Giallo. In the cultivars Tondino, Pearson, and Pantano, naringenin is only present in the peel of control fruits while it is not present in stressed fruits. In contrast, in the cultivars Costoluto and Datterino, naringenin is present only in the peel of the DS group and not in the CTRL. The cultivars Rosso, Pisanello, and Fragola do not show major differences in the concentration of naringenin between control and stressed peels.

## 4. Discussion

Climate change leads to increasingly sudden adverse events that can damage agriculture and the food livelihood of the population. These critical climatic conditions are also likely to affect southern Europe, including Italy [47]. Several physiological, biochemical, and molecular changes occur in plants because of stressful conditions; for example, the scarce availability of water reduces metabolic processes such as photosynthesis [48]. In a previous work we investigated how local tomato cultivars (the same as in the present study) respond to drought in morpho-physiological terms [30]. The previous study found that drought stress causes a decrease in plant growth and photosynthetic efficiency; however, some local cultivars have proven to be tolerant of stress. The Perina and Quarantino cultivars were the most tolerant, with the first cultivar more tolerant in the vegetative phase while the second cultivar was in the reproductive phase. It should be noted that a higher demand for water supply is necessary for tomato plants just at the flowering stage [14] and a water shortage during flowering not only reduces flower development, but also increases their fall [49]. This is also confirmed in the present work, which highlights a fall of flowers, both open and fertilized, and a general delay in their development in stressed plants. For all cultivars, a general decrease in the ripening and developmental time of fruits in stressed tomato plants was observed. The cultivars exhibiting this behavior are expected to complete their life cycle. The different behaviors observed between control (CTRL) and stressed (DS) plants are less evident in the Perina and Quarantino cultivars, where development remains similar between CTRL and DS groups.

Earlier germination is generally observed in stressed samples compared to controls, with the exception of Quarantino, where germination is similar, and Perina, where no germination is observed after four days in both CTRL and DS. After eight days, germination remains similar between CTRL and DS for Quarantino and most cultivars, while Perina shows a lower germination rate in stressed samples. When stress affects the final stage of fruit ripening, germination decreases while no effect is noted when stress acts early in fruit development [15]. Under conditions of environmental stress, it is also well-known that germination is delayed or completely inhibited depending on the intensity of stress and the timing of initiation [50].

Oxidative damage (i.e., the production of reactive oxygen species, ROS) is one of the main consequences of water deficit. Plants have an innate antioxidant system that mitigates the effects of stress and involves the synthesis of antioxidant molecules, as already shown in the cultivars under study [29]. Nevertheless, differences have been found between cultivars so that plants of different genotypes do not implement the same mechanisms and consequently the amounts of antioxidant molecules can be different [29]. Drought-induced oxidative stress does not only have downsides: following stressful conditions, plants can increase the content of antioxidant molecules in fruits, resulting in improved quality and thus benefits to human health. In this study, analyses were performed on the tomato fruit by separating the peel from the pulp, which can have very different concentrations of biomolecules. In the peel, which is normally considered a waste, there is a higher concentration of biomolecules; this is not surprising because the peel is in direct contact with the environment and pathogens/parasites [4]. In general, the data obtained in the present study indicate that total antioxidants increase in the stressed group for most local cultivars, while the stressed group of commercial cultivars often exhibit a decrease in antioxidant concentration compared to controls. Among the stressed cultivars with a higher antioxidant power, both in the peel and in the pulp, the cultivars Perina and Canestrino di Lucca exhibit a high value even in the control group.

Data about total polyphenols reveal a situation like the one outlined for the antioxidant power, with an increase in the stressed group compared to the control. From the analysis of peel, the cultivar with the highest concentration of polyphenols is Perina. Compared to all other cultivars, Rosso di Pitigliano increases the concentration of polyphenols in the stressed group. A lower concentration of these compounds in the stressed group is shown in the cultivar Quarantino, both in the peel and in the pulp; it should be remembered that this cultivar tolerated better drought stress during the reproductive phase. A countertrend observed with respect to other compounds is the flavonoid content. The CTRL group of Quarantino is the cultivar with the highest concentration in the peel while Datterino and Perina are those with the highest concentration in the DS group. Thus, from the data of the present study, it is possible to state that the increase in the above molecules varies among cultivars, in agreement with other work in the literature. For example, work on *Cucumis melo* L. showed that antioxidant power is affected by genotype [51]. In our case, the cultivars with a marked increase in antioxidant molecules are Perina and Rosso di Pitigliano.

The most abundant compounds present in tomato fruits are flavonoids, such as rutin, quercetin, naringenin and caffeic acid, and vitamin C while the most abundant carotenoid is lycopene [2]. These compounds have beneficial effects on human health; in fact, several studies confirm that lycopene plays a role in the prevention of prostate cancer and cardiovascular disease. This is because lycopene may have an inhibitory effect on cholesterol synthesis and may increase the degradation of LDL [52]. In this study, lycopene content in the peel was much higher than in the pulp, but most cultivars decrease lycopene content due to irrigation conditions. There is conflicting data in the literature on this topic. Riggi [53], Atkinson [54], and Klunkin [55] found that drought stress lowers lycopene content compared to well-watered plants. In contrast, Theobald [56] stated that lycopene content increases by more than 27% in drought-stressed fruits. An increase in lycopene content has also been found in tomato fruits grown in southern Italy [57].

Vitamin C is a potent antioxidant that contributes to immune defense by supporting various cellular functions of the innate and adaptive immune systems. Vitamin C also promotes oxidative scavenging activity in the skin, thereby protecting cells from oxidative stress [58]. In our work, there is no increase in vitamin C in stressed plants compared to controls for most cultivars, as with lycopene. This behavior agrees with Seminario [59], where drought stress was shown to cause a reduction in ascorbic acid biosynthesis in soybean plants. The data are also in agreement with Shao [60], in which no increase was reported in tomatoes after drought stress. Other studies have shown that vitamin C increases in relation to water depletion, especially during fruit ripening [57,61], although the magnitude of this effect may also be cultivar dependent [62]. The vitamin content values found in these cultivars are comparable to those described by Ilahy [63].

Rutin is important for several pharmacological activities, including antioxidant, cytoprotective, vasoprotective, anticarcinogenic, neuroprotective, and cardioprotective activities [64]. The analysis of rutin, naringenin and caffeic acid in this study revealed that their concentration in the pulp is extremely low, if not completely absent. In general, it turned out that the exocarp (peel) is the part where these molecules are most abundant. This was expected, since the peel is the part of the fruit most exposed to environmental stresses. The results showed that cultivars behave very differently from each other, with the content of rutin, naringenin, and caffeic acid depending on both genotype and stress conditions. These differences can be attributed to the genetic biodiversity of the cultivars investigated. Perina contains the highest concentration of caffeic acid and naringenin, and large amounts of rutin (highest among controls). For most cultivars, the concentration is higher in stressed fruits than in the control. On the whole, the reported values are higher than those found in the literature [65].

The results of the present work show general agreement with those of Klunklin and Savage [55], i.e., different tomato crops respond differently and therefore generate different concentrations of metabolites when affected by abiotic or biotic stress. Tomato peel, which is much more enriched in bioactives than pulp, is usually considered a waste by processing industries. Actually, the data contained in this work and others indicate that it could be recycled and valorized. In support of this is to mention the recent work of Grassino et al. [66], in which the authors propose the exploitation of the peel for the recovery of bioactives. Approximately 8.5 million tons of peel waste is discarded globally by tomato processing industries; however, valuable bioactive constituents such as lycopene would allow for the revalorization of tomato byproducts that could be incorporated into functional foods [67].

## 5. Conclusions

The experimental evidence in this work showed that, in the absence of drought, Perina is the tomato cultivar with the highest antioxidant power and polyphenol content. On the other hand, the cultivar Quarantino is characterized by high content of total flavonoids in control and lycopene and vitamin C in stressed plants. It is worth noting that Perina and Quarantino show, although with differences, the best response to drought. In particular, Quarantino responds better to stress during the reproductive phase. This suggests that specific Tuscan tomato cultivars may be more suitable for a correct management of irrigation water without affecting natural resources and contributing to a sustainable agriculture. The second prospective concerns bioactive phytochemicals, such as sterols, carotenes, and polyphenols extracted from tomato by-products that could be useful in formulating functional foods and to prevent diseases (such as cardiovascular and Alzheimer’s). In fact, the processing waste (peels) of tomato subjected to drought could have an antioxidant action even at low concentrations by permeating intact through the intestine once integrated into the diet.

## Data Availability

Data available on request due to restriction.

## Supplementary Materials

The following supporting information can be downloaded at: https://www.mdpi.com/article/10.3390/foods11030270/s1. Figure S1: The bar chart illustrates the flowers and fruits development during drought stress. The colors of the bars in the graphics correspond to the different colored strings with which the plants of each cultivar were marked at the beginning of stress (t_0_): PINK for open flowers (OF), BLUE for fertilized flowers (FF), GREEN for small green tomatoes (SGT), RED for large green tomatoes (LGT). The heights of the bars correspond to the number of flowers and fruits at each developmental stage t_0_, t_1_ (10 days) and t_2_ (20 days). The values are expressed as percentage of the total number at t_0_. See text for further details.
