# Supplementary figures and images for: Italian Tomato Cultivars under Drought Stress Show Different Content of Bioactives in Pulp and Peel of Fruits
Source: Foods. 2022 Jan 20;11(3):270. doi: 10.3390/foods11030270 (PMC8834277; doi:10.3390/foods11030270)

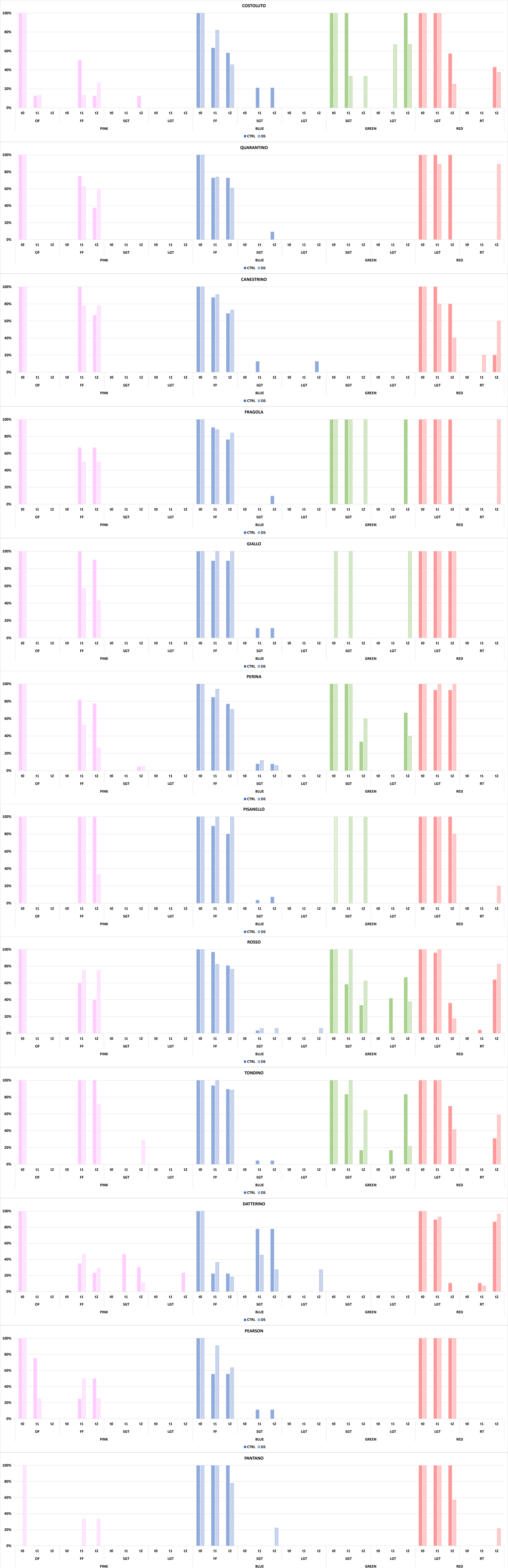

Supplement: Supplementary file 1 [file foods-11-00270-s001.zip › foods-1511229-supplementary.pdf]
